# The Ecology of Salicylic Acid Signaling: Primary, Secondary and Tertiary Effects with Applications in Agriculture

**DOI:** 10.3390/ijms20235851

**Published:** 2019-11-21

**Authors:** Camila C. Filgueiras, Adalvan D. Martins, Ramom V. Pereira, Denis S. Willett

**Affiliations:** Department of Entomology, College of Agriculture and Life Sciences, Cornell AgriTech, Cornell University, Geneva, NY 14456, USA; adm285@cornell.edu (A.D.M.); rvp34@cornell.edu (R.V.P.); deniswillett@cornell.edu (D.S.W.)

**Keywords:** aboveground belowground, plant defense in agriculture, natural enemies, indirect interactions, indirect effects, plant mediated interactions

## Abstract

The salicylic acid pathway is one of the primary plant defense pathways, is ubiquitous in vascular plants, and plays a role in rapid adaptions to dynamic abiotic and biotic stress. Its prominence and ubiquity make it uniquely suited for understanding how biochemistry within plants can mediate ecological consequences. Induction of the salicylic acid pathway has primary effects on the plant in which it is induced resulting in genetic, metabolomic, and physiologic changes as the plant adapts to challenges. These primary effects can in turn have secondary consequences for herbivores and pathogens attacking the plant. These secondary effects can both directly influence plant attackers and mediate indirect interactions between herbivores and pathogens. Additionally, stimulation of salicylic acid related defenses can affect natural enemies, predators and parasitoids, which can recruit to plant signals with consequences for herbivore populations and plant herbivory aboveground and belowground. These primary, secondary, and tertiary ecological consequences of salicylic acid signaling hold great promise for application in agricultural systems in developing sustainable high-yielding management practices that adapt to changing abiotic and biotic environments.

## 1. Introduction

Agricultural productivity is a global priority [1]. With a growing population and increasingly dynamic climate, there has been an intense focus on genetic improvement of food crops for human consumption [2]. Much of this effort has been focused on directly increasing yield under diverse abiotic and biotic conditions. While yields have increased substantially, especially after the green revolution, yield improvements have been stagnating in critical areas [2,3].

As a consequence of these genetic improvement efforts, modern cultivars have lost the adaptability [4] and defenses inherent to their wild ancestors [5,6,7,8]. While this increased productivity, in many cases the modern cultivars can be more susceptible to attack by pests and pathogens [5,6,7,8]. Plants respond to these attack in different ways, defending themselves both directly through physical and chemical defenses against herbivores and pathogens, and indirectly by, for example, recruiting natural enemies of herbivores [9].

These direct and indirect defenses are regulated through biochemical pathways that rely on plant hormones to mediate physiological changes that aid in plant defense [10]. These changes can be genetic involving alterations to transcription and translation, metabolomic affecting synthesis of secondary metabolites, and volatilomic inducing release of volatile signals. While there are a few principal plant defense pathways primarily responsible for defense against pests and pathogens, such as the jasmonic acid (JA) pathway, the salicylic acid (SA) pathway garnered substantial interest for its role in regulating defenses, its inducibility, and potential applications for applied agricultural management in the field [11,12].

Because of these reasons, the SA pathway is the focus of this review. It is important to keep in mind, however, that these systems of plant defense do not occur in isolation. There is ample evidence of cross-talk between plant defense pathways with the JA pathway, for example [13,14,15]. These interactions are usually reciprocally antagonistic; for example, SA can transcriptionally control JA signaling [16,17].

Keeping in mind the potential for cross-talk, this review will focus primarily on the ecology of the SA pathway. Much work has been done elucidating the individual steps in synthesis, mechanisms of induction, and biochemical pathways that form the SA pathway. This review will touch on many of those points, but with a focus on how those pathways and reactions effect communication with the plant itself and with other organisms. The ecology of the SA pathway—how the SA pathway in a given plant mediates interactions between and with other organisms—is just beginning to be understood. The goal of this review is to provide a basis for future work that aims to explore this space more fully.

**Figure 1:**
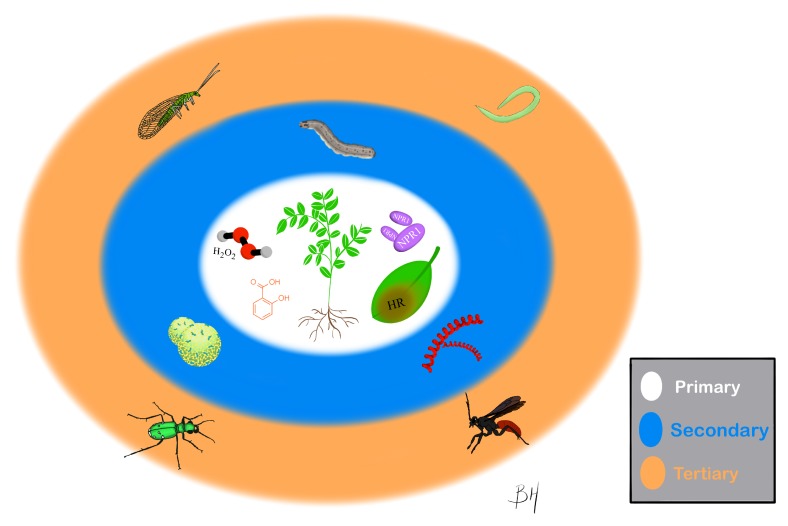
Primary, Secondary and Tertiary Effects of SA Signaling. Primary effects encompass plant specific effects. Secondary effects involve direct and indirect interactions with pathogens and herbivores. Tertiary effects comprise interactions with natural enemies.

To that end, this review will be structured with separate sections focusing on the primary, secondary, and tertiary effects of inducing the SA pathway. The objective of these sections is to highlight primary effects of SA on the plant, the secondary effects of SA on pests and pathogens, and the tertiary effects of SA on natural enemies (Figure 1). In each of those sections, this review will lay the groundwork for what has been done in the area while pointing out opportunities for further work into the ecology of these different effects. The review will close with a discussion of a relatively new advancements and an exciting area of active research: use of the SA pathway for applied control of agricultural pests and pathogens with a discussion of costs and benefits of this approach for plants and managers in applied agricultural systems.

## 2. Primary Effects of SA

Salicylic acid, as a molecule, has remarkable properties in multiple fields [18]. Independent of its rich and storied medical history and modern relationship with aspirin, salicylic acid is a common, nearly ubiquitous, phenolic secondary metabolite of plants [18,19,20]. The chemical properties of the molecule make it readily soluble inside plant tissue [20] and easily transported in its methylated form [21,22]. Its methylated form, methyl salicylate (MeSA), is readily volatilized and, in addition to its role as a cues for other organims, is used in oil of wintergreen [23].

### 2.1. Production and Processing

Production of salicylic acid occurs in plant plastids where the end product in the shikimic acid pathway, chorismic acid can be further processed into either isochorismic acid or prephenic acid then L-phenylalanine and trans-cinnamic acid [19]. These two parallel pathways each rely on separate enzymes, isochorismate synthase (ICS) and phenylalanine ammonia lyase (PAL, responsible for conversion of L-phenylalanine to trans-cinnamic acid) respectively [19,24]. Genes responsible for production of these enzymes and their homologs were identified in several plant species [19,24]. Further modification of salicylic acid to its methylated form can be mediated by BA/SA carboxyl methyltransferase 1 (BSMT1) identified through work in *Arabidopsis* [24].

Whether production of salicylic acid by either the ICS or PAL pathways has ecological consequences for induction of the pathway or downstream effects on other organisms remains unclear. Previous work showed that the ICS pathway may be primarily responsible for production of SA [25], but both pathways were implicated in plant responses to abiotic stressors such as UV exposure and biotic stressors such as pathogen infection [24]. Much of the work exploring these pathways was done in *Arabidopsis* model systems [24,26]. To our knowledge, and lacking from recent reviews, no work has been done exploring differential effects of pathogen and pest stimulation of the IC and PAL pathways for salicylic acid biosynthesis either in *Arabidopsis* or other plant systems [19,24,25,26,27].

Following production of SA, the plant hormone can be modified in several different ways that affects its solubility, mobility, and use as a signal. Among other steps that may occur post-synthesis, SA can be glucosylated, methylated, and conjugated with amino acids [19,24,25]. Glucosylatation of SA via glucosyltransferases convert SA to a relatively non-toxic deactivated form that can can be stored long-term in cell vacuoles [24]. Methylation of SA to form MeSA creates a highly mobile signal with a host of ecological effects [19,24,25]. Amino acid conjugation is an active area of research and is likely involved in degradation of SA [24]. These three forms of SA modification-storage, transport, and degradation-among others are important means by which which plants regulate levels of SA post-production and mediate effects on plant physiology.

Regulation of SA is almost universally important for plant physiology, but not universally uniform; plants vary substantially in basal levels of SA [28,29]. Multiple order of magnitude differences between species were recorded, even within the same family [28,29]. Basal levels of SA in *Arabidopsis* range from 0.250 μg to 1 μg g${}^{-1}$ FW [28]. Within a given plant levels of SA can also vary widely. Shoots and roots can have wildly different amounts of SA both basally and as a result of the physiologic responses of different plant tissues [28,30].

### 2.2. Plant Response

Downstream of production, SA can have a multitude of effects on plant physiology in all parts of the plant. SA affects plant thermogenesis, stomatal dynamics, seed germination, cell growth, vegetative growth, flowering, photosynthesis, responses to abiotic stresses and defensive responses against pests and pathogens [19,29,31,32,33,34,35,36,37,38].

In mediating responses to abiotic stress, SA increases the efficiency of the antioxidant system in plants [32]. SA can lower levels of reactive oxygen species preventing cell damage from free radicals and promoting intracellular redox homeostasis [32,39]. This increased tolerance to oxidative stress also plays a role in mediating interactions with other organisms; reactive oxygen species are involved in cell death responses and generation of long-term resistance to pests and pathogens [32,40]. Of particular importance to the ecology of the SA pathway, induction of the SA pathway can result in localized and systemic defense responses within the plant.

When plants are attacked by microbes and pathogens, a series of plant defense responses can be triggered. Pattern recognition receptors in the plant can detect pathogen and microbe specific patterns that can, in turn, stimulate pattern triggered immunity (PTI) [41,42,43]. If PTI is suppressed by pathogen effectors, plants can rely on an additional level of defense in effector triggered immunity (ETI) [37,42,43]. These defenses can result in programmed cell death at the site of infection regulated by specific plant resistance genes and termed the hypersensitive response [43,44]. SA was implicated in mediating both PTI and ETI responses in monocots and dicots and is a necessary component of systemic acquired resistance [37,43,45,46].

Systemic acquired resistance (SAR), the ability of plants to develop long-term resistance to micro-organisms even in parts of the plant not initially attacked, is a key aspect of plant defense and dependent upon accumulation of SA [45,46]. SA can increase amounts of pathogenesis-related (PR) proteins with anti-microbial properties through systemic changes to transcriptional programming via interaction with transcriptional cofactors of Non-expresser of PR genes (*NPR*) [37,47,48,49,50,51]. Importantly, while SA is an important and necessary component of SAR, it is not the mobile signal for induction. Methyl salicylate, azelaic acid, pipecolic acid, and its derivative N-hydroxypipecolic acid among others, all play a role as mobile, and some cases, volatile signals for systemic acquired resistance [52,53,54,55,56].

### 2.3. Induction

While induction of the SA pathway by pathogens can result in SAR, activation of the SA pathway can be induced exogenously through application of elicitors and plays important roles in regulating responses to other organisms including attack by other plants, and by insect herbivores.

Ever since early recognition of SA as a plant signal, exogenous application of SA has been used to induce adaptive responses in plants to both abiotic and biotic stress [32,39,57]. Induction of SA pathway with exogenous elicitors was successfully conducted using a variety of compounds including SA proper, MeSA, and Benzothiadiazole (BTH, benzo(1,2,3) thiadiazole-7-carbothioic acid S-methyl ester, a synthetic analog of SA), and S-methylmethionine salicylate, among others [58]. Exogenous application of these elicitors through spraying or seed treatment can often mimic adaptations to abiotic and biotic stress, particularly defense responses triggered by pathogenic micro-organisms, plants, and insects [12,58,59,60,61].

Plants attacked by other parasitic plants can exhibit pathogenically similar responses with regulation by SA [62]. *Striga* infection in *Sorghum* can elicit hypersensitive responses at sites of attack [63]. Similarly, Dodder infection in alfalfa can induce expression of PR genes for defense [64]. Dodder attacks on tomato induced hypersensitive-like responses and elevated SA levels along with other plant hormones involved in defense [65]. This is still an active area of research; the mechanisms, elicitors, and ramifications of SA induction by plant parasites is still being explored [62].

Given the prominent role of SA in regulating plant responses to other forms of attack, it should come as little surprise that feeding by insect herbivores can also induce SA mediated effects for plant defense. While SA is relatively better explored in the context of pathogen defense, insects-particularly sucking insects-can also elicit responses. Aphid feeding by the green peach aphid (*Myzus persicae*) on *Arabidopsis* induces SA related gene expression involved in pathogen defense [66]. Feeding by the Russian wheat aphid (*Diuraphis noxia* Mordvilko) on wheat induces SA accumulation which plays a role in resistance [67]. Likewise, tomato resistance to potato aphid mediated by the Mi-1 gene relies on SA and is a case study for similar plant defense responses to plant pathogens and sucking insects [68]. Such responses can even alter activation of other plant defense pathways; silverleaf whitefly (*Bemisia tabaci* type B) feeding on *Arabidopsis* induces the SA related plant defenses while suppressing the JA defense pathway [69].

Additionally, there is some evidence that SA may be involved in defense against chewing insects. While many studies of plant defense pathway induction by chewing insects point to the role of the JA pathway [70] in mediating chewing herbivore responses, SA was implicated in plant defenses of tomato in response to feeding by larvae of the cotton bollworm (*Helicoverpa armigera* Hubner) [71].

Induction of the SA acid pathway in response to attack by plants, pathogens, and pests is not a given, however. Other factors can play a role. Endogenous levels of other plant hormones can be crucial in determining whether and how SA related responses develop within a plant [17,72]. Ethylene, for example, not only can interact with SA responses, but also affect JA-SA crosstalk [72,73]. Circadian rhythms and presence of light can be important in regulating responses affecting both the attacker and how the plant responds to pathogens and herbivores [74,75,76].

## 3. Secondary Effects of SA

### 3.1. Direct Effects

Induction of the SA pathway has a direct role in mediating interactions with and between pathogens and herbivores. As was touched on briefly, activation of SA related plant defenses can result in production of PR proteins and induction of SAR with negative consequences for infecting pathogens [26,37,45,49]. Viral, bacterial, fungal, and oomycete pathogens can all be negatively impacted through induction of SA associated resistance in both monocots and dicots [77,78]. Specifically, SA plays an important role in resistance to tobacco mosaic virus in tobacco [46], resistance to *Pseudomonas* in *Arabidopsis* [79], resistance to *Alternaria* fungus in potato [80], rice blast fungus in rice [81], and defense against *Phytophthora infestans* in potato [82]. In addition to halting pathogen infection, induction of SAR may render a plant inaccessible for future pathogen attack by altering patterns and distributions of pathogen infection locally. These effects can even cascade to alter community structures of microorganisms [83].

Community effects of SA mediated plant defenses are not only limited to effects at the micro-organism community level, but also extend to other plants. Volatile communication between plants can cause cascading effects of SA induction in plant communities by triggering SA related defenses in neighboring plants [84,85]. This phenomenon was best studied in willow and sagebrush systems [86,87] and relies on transmission of plant volatiles such as MeSA among others aboveground [84,88]. Communication can also take place belowground [88]; recent work has indicated a role for SA signaling, among other plant defense pathways, in belowground plant-plant communication [89,90,91].

Similarly, SA induction can have negative consequences for the fitness of attacking herbivores. As a defensive molecule, phenolic compounds such as SA can act as deterrents and be toxic to insect herbivores [92]. In willows, for example, SA and related compounds play a role in reducing performance of generalist chewing insects such as *Manduca* and *Operophtera* [92,93]. Also in willows, SA mediates resistance development against a gall midge that produces a characteristic hypersensitive response resulting in reduced insect larval survival [94].

SA induction can have additional direct effects against insect herbivores. In *Arabidopsis*, egg deposition and larval feeding by *Pieris brassicae* can interact to induce higher SA levels [95]. SA can accumulate at sites of oviposition [96] and larval feeding can exacerbate this effect [95]. At the same time, there is enhanced expression of PR genes [95]. Larvae feeding on egg-induced plants perform poorly and gain less weight [95]. Importantly, *Arabidopsis* mutants deficient in SA pathway components do not show the same larval effects [95]. SA not only affects larval performance of *P. brassicae* in *Arabidopsis*, but also potentially affects oviposition behavior; MeSA tends to deter oviposition by *P. brassicae* when either dispensed exogenously or expressed constitutively at high levels [97]. Similar effects on oviposition and performance were observed to some extent in other systems, but either been tested solely through exogenous application or not explored to the same extent [98,99].

Fitness effects of SA induction are not always negative for the offending herbivore, however. In tobacco plants infected with the tobacco mosaic virus, over-expression of PAL increases SAR to the tobacco mosaic virus while under-expression weakens it [100]. The inverse is true for insect herbivory; plants with a weak SAR response were better able to fend off herbivory while plants with a strong SAR response were not able to defend as well against herbivory by *Heliothis virescens* larvae [100]. This phenomenon was observed to some extent in other pathosystems and is mediated by cross-talk and trade-offs between plant defense pathways [100,101]. As mentioned in the introduction, the SA pathway does not act in isolation but can and often interacts with other plant signalling systems, such as the JA pathway.

### 3.2. Interactions

The preceding example is one prominent case of the SA pathway mediating indirect interactions between plant pathogens and insect herbivores. The ecological consequences of these indirect interactions are only beginning to be understood, but exist in numerous study systems. Interactions can go both ways; in the above example, SA was shown to play a role in pathogen resistance affecting herbivore feeding but herbivore feeding can also impact pathogen infection. Feeding by *Helicoverpa zea* larvae on tomato leaves caused a reduction in infection by the bacterial pathogen *Pseudomonas syringae* [102]. While SA has not always been implicated in these interactions, the role of plant defense pathways in mediating many forms of indirect interactions was excellently reviewed [77] and can occur even across the aboveground-belowground divide [103]. The mechanisms mediating these interactions are still being explored, but sequence of infection or attack is incredibly important as are the identities of the pathogens, plants, and herbivores involved in the interaction [104,105,106].

### 3.3. Temporal Considerations

The role of the SA pathway in mediating ecological effects and interactions between other organisms has an inter-generational temporal component as well. There are epigenetic effects of SA mediated plant defense signaling [19]. DNA methylation and histone modification can play a role in mediating plant defenses through regulation of plant defense genes and affecting SAR [19,107,108]. There is also limited evidence that some of these epigenetic modifications can be heritable in both *Arabidopsis* and bean [107,109,110,111]. The ecological ramifications of epigenetic effects of SA remain to be explored but one can imagine a situation where induction of the SA pathway not only has a priming effect on the plant during its lifetime perhaps resulting in SAR, but also has inter-generational effects that affect pathogen and herbivore populations long term.

## 4. Tertiary Effects of SA

Just as induction of the SA pathway has primary metabolic consequences for the plant and secondary consequences for other organisms such as pests and pathogens, induction of the SA pathway can have tertiary effects on additional trophic levels affecting behavior and recruitment of natural enemies both aboveground and belowground.

### 4.1. Aboveground Natural Enemies

Since early work showing the role of herbivore induced plant volatiles in recruiting natural enemies aboveground [112], the role of SA has been explored in influencing natural enemies of plant herbivores. Natural enemies can be predators or parasites of plant herbivores and range from specialist parasitic wasps to generalist predatory beetles. Natural enemies of insect herbivores, particularly parasitic wasps, are known to respond to a variety of cues released from plants and insects in order to locate their future hosts and can learn to respond to a variety of dynamic and ephemeral cues [113,114].

One prominent cue that can be directly linked to induction of the SA pathway is the volatile methyl salicylate (MeSA). MeSA is a phenolic signal produced from SA and is involved in plant-plant communication as a mobile and volatile signal for systemic acquired resistance [23,53]. MeSA in its role as a plant defense signal is likely conserved [115]; many plants release MeSA as a component of herbivore induced plant volatile blends in response to feeding by insect herbivores (Table 1).

Predators and parasites of these insect herbivores can also perceive and respond to MeSA. Indeed, MeSA has been shown to be attractive to a wide range of insect natural enemies ranging from micro-hymenoptera to lacewings in laboratory and field studies (Table 2). In many cases, recruitment of these natural enemies in response to release of MeSA can occur over relatively large distances and reduce pest populations. It bears noting, however, that despite the apparent broad use of MeSA as a beneficial plant signal resulting in the attraction of predators and parasites to reduce herbivore feeding, this is not universally true. In trials comparing *Arabidopsis thaliana* plants compromised in the production of MeSA with wild-type plants, MeSA compromised plants were more attractive to parasitoids, natural enemies of biotic stressors, than their wild-type counterparts releasing MeSA [125].

### 4.2. Belowground Natural Enemies

While the SA pathway plays a significant role in regulating plant defenses and mediating interactions with herbivores and natural enemies aboveground, its role in belowground indirect defenses is just beginning to be understood. Plants inhabit two distinct environments; just as the shoots and leaves of plants aboveground can benefit from recruitment of natural enemies to reduce aboveground herbivore pressures, so too can plant roots. Belowground herbivory is an important factor affecting plant performance, if relatively unexplored [134]. New methodologies, technologies, and approaches have been opening up the frontier of belowground plant defense interactions in recent years [135,136] with discoveries that highlight the importance of SA in belowground interactions with natural enemies.

In addition to adapting to two distinct environments, plants must also contend with an embedded corollary; natural enemies belowground are inherently different than those aboveground. Parasitic wasps tend to be less effective against belowground herbivores. Instead, natural enemies belowground can include entomopathogenic nematodes and soil-dwelling mites. Similar to aboveground systems, belowground feeding by root herbivores can induce release of volatiles that recruit predatory mites [137] and entomopathogenic nematodes [138,139,140]. These entomopathogenic nematodes effect the death of their insect hosts with the aid of symbiotic bacteria [141]. Release of herbivore induced plant volatiles belowground has been shown to reduce herbivory [142,143] and can increase probability of pest insect mortality by approximately 90% [144].

The role of the SA pathway in mediating these changes is just beginning to be explored. Exogenous application of MeSA in citrus and corn plants can cause recruitment of entomopathogenic nematodes [132,133]. In citrus, this relationship has been explored further; exogenous application of MeSA can induce release of the terpene volatile limonene which is attractive to entomopathogenic nematodes [133]. Effects of SA induction on natural enemies belowground could potentially have far reaching consequences. Trials examining distances of recruitment suggest that release and diffusion of these volatile signals can attract beneficial natural enemies from distances as great as 60 cm in sandy soil types [145].

### 4.3. Connecting Aboveground and Belowground with SA

This work suggests a specific role for SA in not only connecting belowground and aboveground plant systems, but also in mediating tertiary effects between aboveground organisms and belowground natural enemies and vice versa. Indeed, recent work showed that belowground feeding by insect larvae can induce release of volatiles aboveground attractive to parasitoids of the adult insect [146]. While no plant defense pathway was implicated in that work, the ability of induction of the SA pathway to effect release of terpene volatiles and recruit entomopathogenic nematodes belowground suggests a broad role for the SA pathway facilitating tertiary effects by communicating with and connecting natural enemies below and aboveground.

## 5. Applications of SA Induction for Control of Pests and Pathogens

Induction of the SA pathway has clear ramifications for plants, pathogens, herbivores, and natural enemies above and belowground with ecological consequences radiating from SA mediated interactions at multiple trophic levels. Observation of these types of effects naturally leads to contemplation of possible application in agriculture. Using the SA pathway in agriculture to manage pests and pathogens has generally followed three approaches: (1) exogenous application of compounds that induce the SA pathway for direct plant defense; (2) genetic modification of plants to alter plant defense expression; (3) exogenous application of SA related volatile compounds (e.g., MeSA) to attract natural enemies that control insect pests.

### 5.1. Exogenous Induction

Early investigations into using exogenous applications of SA inducing compounds to elicit plant defenses pathways, induce resistance, and augment SA signaling opened the way for consideration of novel management strategies for control of agricultural pests and pathogens. Exogenous application can take many forms involving a wide range of elicitors and synthetic SA analogs that can result in SAR and enhanced resistance to pathogen load [147,148]. In addition to the examples cited above, exogenous application of MeSA to *Nicotiana benthamiana* increased resistance to challenges by the bacterial pathogen *Pseudomonas syringae* [149]. Repeated application of MeSA strengthened this response [149] suggesting that exogenous application in field settings could hold potential for managing pathogen resistance. Field wide applications of exogenous elicitors may not even be necessary; recent work showed that positive feedback loops involving SAR and monoterpene communication could potentially propagate SAR at the population level using plant–plant communication to magnify spatial effects [150]. Effects of exogenous elicitors could also be magnified through time across generations as epigenetic effects of exogenous elicitors have been documented and could potentially engender inter-generational defense with fewer applications [151].

This approach could not only have appreciable effects on plant disease, but also increase yield. In some crop systems, exogenous application of elicitors can have beneficial effects for crop management comparable to pesticide controls [11]. In several important agricultural crops including monocots and dicots, exogenous induction of SAR against bacterial and fungal pathogens in the field has beneficial effects in reducing disease severity in some cases even exceeding benefits seen by pesticide controls [11]. Importantly for consideration of utility in agricultural settings, disease reduction as a result of SAR induction was in some cases associated with increases in yield [11].

Beneficial outcomes on disease management from exogenous application of SA elicitors are not guaranteed, however. Results can be dependent on the identities of the plant and pathogen species [11]. Fusarium wilt (*Fusarium oxysporum f.sp. cucumerinum*) in cucumber, for example, does not respond to exogenous induction of SAR [152]. Multiple elicitors in peanut also failed to control fungal late leaf spot, even creating undesirable effects and augmenting fungal growth [153]. These undesirable outcomes could reflect a lack of understanding of elicitor mechanisms. Recent work has shown that the SA synthetic analog BTH does not confer resistance to *Rhizoctonia solani*, the causal agent of sheath blight disease in the grain species *Brachypodium distachyon* because it induces genes related to JA signaling [154]. In this same system, exogenous application of SA does confer resistance [154].

Non-beneficial agricultural outcomes of exogenous induction of SA defenses are not limited to lack of pathogen control, however. As discussed above, stimulation of systemic acquired resistance may be beneficial in reducing pathogen load, but may result in crop plants becoming more susceptible to herbivory [155]. There are well documented examples of trade-offs between pathogen and herbivore resistance [101]. Trade-offs also encompass other physiological effects; if a crop plant is allocating more energy and resources to defense, less may be allocated to production. Plant defense pathways and growth regulation are inextricably entwined [156]. In the case of SA, the shikimic acid pathway is a starting point for biosynthesis and also critically important in amino acid production [19]. Optimizing defense and yield trade-offs will continue to be a consideration in applying knowledge of SA defenses in agriculture and may have to be considered on a case by case basis. With certain crops under pathogen pressure, but not insect pressure, exogenous stimulation of plant defenses could increase yields, while in other situations, yield may be suppressed by exogenous applications of elicitors either due to no effect on pathogen resistance, negative effect on herbivore defenses, negative effect on yield investments, or some combination of all three.

### 5.2. Genetic Approaches to Using SA in Agriculture

An ideal solution to address the challenges and trade-offs listed above would be crops that have defenses turned off in situations where pest and pathogen pressure is absent that then are strongly activated in situations where pest and pathogen pressure are prevalent [156]. While this characteristic may have been present to some extent in wild progenitors, domestication of agricultural crops of economic importance can have substantial effects on plant secondary chemistry with potential consequences for the ecology of pest and pathogen interactions [157,158]. Over-expression and constitutive expression of specific genes involved in plant defense pathways can create enhanced and broad spectrum resistance to pathogens [159]. While these approaches might be effective under situations of intense pathogen pressure, they suffer from the same trade-offs and drawbacks discussed in the exogenous induction section; constant allocation of plant resources to defense through genetically modified constitutive expression likely has negative consequences for crop yield.

Recent work shows promise in obviating those drawbacks by developing a switch that would balance defense and production trade-offs [156]. Induction of plant immunity through PTI can result in global translational reprogamming that occurs rapidly following pathogen infection [160]. The genetic elements responsible for that reprogramming can be packaged and inserted into *Arabidopsis* and rice resulting in resistance to agriculturally relevant pathogens across generations while balancing fitness and yield costs [161]. These results hold considerable promise for development and engineering of high-yielding plant varieties adaptive to and successful across wide ranges of pathogen pressure.

### 5.3. Natural Enemy Attraction

While engineering plants that selectively activate SA defense pathways in areas of high pathogen pressure holds considerable promise for defense against plant pathogens, herbivore pressure can also substantially impact yields. The SA pathway has limited efficacy for defense against herbivore pests of agricultural pests. As discussed above, SA can play a role in direct defense against sucking insects such as aphids [66,67,68,69]. A potentially more promising approach for control of insect herbivores through SA related defenses in agricultural systems is via attraction of natural enemies. As mentioned above, release of volatiles related to SA defenses can recruit natural enemies above and belowground and reduce herbivore populations [133] (Table 2). This attraction can be accomplished either through deployment of lures releasing volatiles such as MeSA or through exogenous induction of SA in plants. Meta-analysis of this attraction showed large and relatively invariant effects of attraction across a wide range of predator and parasitoid taxa [12].

The efficacy of this approach on reducing pest populations could be highly variable however and merits further exploration into appropriate means of implementation. Attraction of large numbers of predators and parasitoids may have non-target effects and does not necessarily result in improved pest control; attracted natural enemies may not necessarily be effective in controlling the offending pest species for several reasons including phenology (pest life stage plays an important role in susceptibility), relative population densities, and competing hosts [162]. Additionally, long term release of attractive signals such as MeSA could diminish in efficacy over time, particularly if used prophylatically in the absence of abundant pest populations. Natural enemies responding to a volatile cues in the absence of host resources will learn to avoid this deceptive signal potentially to the chagrin of agricultural producers hoping for continuous natural enemy protection.

### 5.4. Opportunities

Knowledge of SA related defense signaling and potential applications to agricultural challenges burgeoned in recent years. While there is substantial work that remains to be done in understanding basic mechanisms behind primary, secondary, and tertiary ecological effects of SA signaling, what work that has been done points to numerous opportunities for developing methods that enhance sustainable production of important agricultural crops through efficient and efficacious management of abiotic and biotic challenges. These contributions could engender a second green revolution: a plant defense-based revolution-leveraging intelligent adaptations to abiotic and biotic challenges to preserve and increase sustainable crop yields and feed a hungry planet. 

## Figures and Tables

**Table 1:** MeSA released from listed plant species in a blend of herbivore released plant volatiles after feeding by listed herbivore species.

	Plant		Herbivore	
Family	Species	Common Name	Species	Citation
Fabacae	*Phaseolus lunatus*	Spider Mite	*Tetranychus urticae* Koch	[116]
Fabacae	*Phaseolus lunatus*	Spider Mite	*Tetranychus urticae* Koch	[117]
Solanaceae	*Solanum lycopersicum*	Spider Mite	*Tetranychus urticae* Koch	[118]
Cucurbitaceae	*Cucumis sativus*	Spider Mite	*Tetranychus urticae* Koch	[119]
Brassicaceae	*Brassica oleracea capitata* L.	Garden Whites	*Pieris* spp.	[120]
Rosaceae	*Pyrus communis*	Pear Psyllid	*Psylla pyricola* Forster	[121]
Cannabaceae	*Humulus iupulus* L.	Damson Hop Aphid	*Phorodon humuli* Forster	[122]
Rosaceae	*Prunus padus*	Bird Cherry–Oat Aphid	*Rhopalosiphum padi* L.	[123]
Solanaceae	*Nicotiana attenuata* Torr. ex Wats	Five-Spotted Hawkmoth	*Manduca quinquemaculata*	[124]
Solanaceae	*Nicotiana attenuata* Torr. ex Wats	Leaf Bug	*Dicyphus minimus*	[124]
Solanaceae	*Nicotiana attenuata* Torr. ex Wats	Tobacco Flea Beetle	*Epitrix hirtipennis*	[124]
Fabaceae	*Lotus japonicus*	Spider Mite	*Tetranychus urticae*	[117]

**Table 2:** Natural Enemies Recruited by MeSA.

Plant		Herbivore		Natural Enemy				
Family	Species	Common	Species	Common	Species	Impact on Herbivore	Setting	Citation
Cannabaceae	*Humulus lupulus*	Damson-Hop aphid	*Phorodon humuli* [122]	Green Lacewing	*Chrysopa nigricornis*	Not Reported	Field	[126]
Fabaceae	*Phaseolus lunatus*	Spider Mite	*Tetranychus urticae*		*Phytoseiulus persimilis*	Not Reported	Lab	[127]
Fabaceae	*Glycine max* L.	Soybean Aphid	*Aphis glycines*	Green Lacewing	*Chrysopa nigricornis*	Reduced	Field/Lab	[128]
Fabaceae	*Glycine max* L.	Soybean Aphid	*Aphis glycines*	Syrphid Flies		Reduced	Field/Lab	[128]
Cannabaceae	*Humulus lupulus*			Ladybeetle	*Stethorus punctum picipes*	Not Reported	Field	[129]
Cannabaceae	*Humulus lupulus*			Minute Pirate Bug	*Orius tristicolor*	Not Reported	Field	[129]
Cannabaceae	*Humulus lupulus*			Bigeyed Bug	*Geocoris pallens*	Not Reported	Field	[129]
Cannabaceae	*Humulus lupulus*			Syrphidae		Not Reported	Field	[129]
Cannabaceae	*Humulus lupulus*			Empididae		Not Reported	Field	[129]
Cannabaceae	*Humulus lupulus*			Sarcophagidae		Not Reported	Field	[129]
Cannabaceae	*Humulus lupulus*			Agromyzidae		Not Reported	Field	[129]
Cannabaceae	*Humulus lupulus*			Micro-hymenoptera		Not Reported	Field	[129]
Vitaceae	*Vitis labrusca*			Green Lacewing	*Chrysopa nigricornis*	Not Reported	Field	[130]
Vitaceae	*Vitis labrusca*			Lacewing	*Hemerobius* sp.	Not Reported	Field	[130]
Vitaceae	*Vitis labrusca*				*Deraeocoris brevis*	Not Reported	Field	[130]
Vitaceae	*Vitis labrusca*				*Stethorus punctum picipes*	Not Reported	Field	[130]
Vitaceae	*Vitis labrusca*			Minute Pirate Bug	*Orius tristicolor*	Not Reported	Field	[130]
Rosaceae	*Fragaria* sp.	Aphididae, Thripidae, Cicadellidae		Minute Pirate Bug	*Orius tristicolor*	No Effect ^1^	Field	[131]
Rosaceae	*Fragaria* sp.	Aphididae, Thripidae, Cicadellidae		Chrysopidae		No Effect ^1^	Field	[131]
Poaceae	*Zea mays*	Cucurbit Beetle	*Diabrotica speciosa*	Entomopathogenic Nematode	*Heterorhabditis amazonensis*	Not Reported	Lab	[132]
Rutaceae	*Citrus paradisi × Poncirus trifoliata*	Citrus Root Weevil	*Diaprepes abbreviatus*	Entomopathogenic Nematode	*Steinernema diaprepesi*	Not Reported	Lab	[133]

^1^ Most effects were found to be not significant, although a marginal decrease was observed for one year for leafhoppers [131].

## Abbreviations

SA	Salicylic Acid
JA	Jasmonic Acid
PAL	Phenylalanine ammonia lysase
ICS	Isochorismate synthase
MeSA	Methyl Salicylate
SAR	Systemic Acquired Resistance
PTI	Pattern Triggered Immunity
PR	Pathogenesis related
NPR	Non-expressor of PR
ETI	Effector Triggered Immunity
BTH	Benzo(1,2,3)thiadiazole-7-carbothioic acid S-methyl ester
